# Measuring psychosocial factors in health surveys using fewer items

**DOI:** 10.1177/2055102920975983

**Published:** 2020-12-24

**Authors:** Evalill Nilsson, Peter Garvin, Karin Festin, Marika Wenemark, Margareta Kristenson

**Affiliations:** 1 Linköping University, Sweden; 2 Linnaeus University, Sweden

**Keywords:** methodology, quantitative methods, reliability, resources, risk factors, validation

## Abstract

The present study investigated the possibility of reducing length of psychosocial scales, while maintaining validity, using easily manageable techniques. Data were collected 2003–2004 in a Swedish general population; *n* = 1007, ages 45–69, 50% women. Eight psychosocial scales were reduced from 6–20 to 3–7 items maintaining Cronbach’s alpha >0.7 and correlation coefficients between full and reduced scales > 0.85. Relationships to biomarkers for inflammation, self-rated health and 8-year incidence of coronary heart disease showed no difference between full and reduced scales. It was possible, using these easily manageable methods, to reduce scale length without threatening validity for use in population surveys.

## Background

There is a global trend of increased usage of health questionnaires (scales), both in population health surveys and in health care systems, as patient-reported measures. In multipurpose surveys with a need to use several scales on different issues, such as psychosocial factors and health status, these surveys tend to be extensive. Long questionnaires with high respondent and administrative burden have a greater risk of measurement error due to mistakes or ‘satisficing’, that is, respondents finding shortcuts for completing the questionnaire, for example by choosing the same response option for all items, or choosing ‘don’t know’ if such an option is available (Krosnick, 1991; Peytchev and Peytcheva, 2017).

In surveys where a combination of many scales is required to cover all relevant topics, these different scales and all other measures will compete for space in the survey. In such situations, the choice may be between using a short form of a scale or omitting the scale completely. This has led to a request for shorter scales. For some psychosocial and health status scales there are no official short forms, while for others the existing short forms may not be short enough. Sometimes, researchers have instead produced customised short forms to be used in specific contexts (Lundberg and Nyström Peck, 1995; Schumann et al., 2003) or simply employ single-item screening (Reme et al., 2014).

There is often a reluctance to carry out official updates of established scales. There can be many reasons for this, for example, scales may be seen as a gold standard, or be used for direct comparisons with previous studies that used the scales. People may also refrain from creating official short forms or revising a scale since a scale needs to be validated in every new form and for every new context it is used in, and this validation procedure may be considered time consuming (De Vet et al., 2011). Established scales are therefore perhaps not challenged as often as would be beneficial. For example, a scale used in a study conducted 20–30 years after the scale was developed may need to be modernised or revised to fit the language, people and society of today. Though modern, more advanced methods of scale construction may theoretically be the best choice, they require resources not generally available. Hence, less resource-intensive methods and easily manageable technique also need to be available (Kleka and Soroko, 2018).

The aim of the present study was to evaluate the possibility of reducing the length of some legacy psychosocial scales while maintaining validity, using an easily manageable and available technique.

## Methods

### Study population

The present study is based on the study population from the longitudinal study ‘Life conditions, Stress and Health’ (LSH), which investigates to what extent psychosocial factors can explain socioeconomic differences in the risk of coronary heart disease (CHD), and if psychobiological pathways can mediate these associations. Therefore, a large range of psychosocial instruments was included in the LSH study. The participants, aged 45–69 years, were randomly selected from the general population and invited consecutively to reach a study population size of *n* = 1000, evenly distributed by age and sex. All citizens in the given age range and living in any of the catchment areas of ten primary health care centres in the County of Östergötland in south east Sweden at the time of enrolment were eligible for invitation. The participation rate was 62.5%, comprising 502 men and 505 women. Informed written consent was obtained from all individual participants included in the study.

The participants visited their primary health care centre in late 2003 and early 2004 for a brief health examination and for the collection of morning blood samples in a fasting state. In addition, several health questionnaires and psychosocial scales were answered. The sample was representative of the population in terms of educational attainment, employment rates, and immigrant status (Garvin et al., 2015). In 2012 an 8-year registry follow-up study of determinants for incident CHD was performed (Lundgren et al., 2015).

### Psychosocial scales

Several different legacy psychosocial scales were included in the present study, covering both intrinsic psychological resources and psychological risk factors (known to be associated with risk of somatic disease, for example CHD), and extrinsic social factors (social support), which together covered a broad spectrum of psychosocial domains. Hence, when referring to the scales as a group in the present study, the term psychosocial is used.

Selected scales in the present study are: (1) Psychological resources: Mastery, Self-Esteem, Sense of Coherence (SOC), (2) Psychological risk factors: Centre for Epidemiological Studies Depression Scale (CES-D), Vital Exhaustion, Cook-Medley Hostility Scale (Cynicism sub scale) and (3) Social support: Availability of Social Integration (AVSI) and Availability of Attachment (AVAT). Below follows a brief description of the scales.

#### Mastery

Mastery aims to capture the ability to cope with ‘social experiences that adversely penetrate people’s emotional life’, and includes items concerning the extent to which one regards one’s life chances as being under one’s control in contrast to being fatalistically governed (Pearlin and Schooler, 1978).

#### Self-esteem

Self-esteem is based on Pearlin’s adaptation of Rosenberg’s Self-Esteem Scale and aims to capture the positiveness of one’s attitudes towards oneself (Pearlin and Schooler, 1978).

#### Sense of coherence

SOC describes a perception that life is seen as meaningful, comprehensible and manageable, which has been shown to contribute to good health despite harsh conditions sometimes present in daily life (Antonovsky, 1987). SOC is available in several versions; in the present study, the 13-item version was used.

#### CES-D

The CES-D scale was designed to measure depressiveness in a normal population, capturing the major components of depressive symptomatology with an emphasis on the affective component, and is not to be used as a diagnostic tool for clinical depression (Radloff, 1977).

#### Vital exhaustion

Vital exhaustion measures a person’s perception of both mental and physical fatigue. A slight modification of the Maastricht Vital Exhaustion Questionnaire (Appels et al., 1987), based on 19 items instead of the original 21, was used in the present study.

#### Cynicism

Cynicism is one of the subsets derived from the Cook-Medley Hostility Scale (Barefoot et al., 1989). The items are focused on perception of others’ behaviour in everyday life, and aim to capture a negative view of humankind, viewing people in general as unworthy, deceitful and selfish.

#### AVSI and AVAT

These two scales have their origin in the comprehensive Interview Schedule for Social Interaction (ISSI). AVSI concerns the number of people that the respondent interacts with in daily life during a week and their social network, that is, addresses the quantity of social ties. AVAT (also called emotional attachment or emotional support) concerns what a person’s social network provides in terms of emotional support, that is, addresses the quality of social ties (Undén and Orth-Gomér, 1982).

### Item reduction procedure

To shorten the psychosocial scales, a reduction procedure was performed based on internal consistency analyses, removing one item at a time. After re-coding of reversed items, Cronbach’s alpha coefficients were calculated for each full scale, and thereafter for all reduced versions of each scale after the removal of one item (e.g. a full scale with 10 items yielded 10 different nine-item scales). For each scale, the reduced version that received the highest Cronbach’s alpha went on to the next step, where the procedure was repeated (i.e. the nine-item scale from the example above with the highest alpha value was used to yield nine different eight-item scales). Repetition commenced as long as the reduced scales retained Pearson intercorrelation coefficients ⩾0.85 with the full scales and Cronbach’s alphas ⩾0.70, and the absolute difference in Cronbach’s alpha compared with the full-scale was <0.1. The minimum number of items left in a reduced scale was set to three.

### Validation measures

#### Biomarkers

Two biomarkers of inflammation, Interleukin-6 (IL-6) and Matrix metalloproteinase-9 (MMP-9), with known relationships to psychosocial factors (Marteinsdottir et al., 2017), were used in this study. Blood samples were collected from the participants during their visit to their primary health care centre and stored in a biobank. Biochemical analyses were performed as described in Marteinsdottir et al. (2017). In short, IL-6 and MMP-9 were measured in EDTA plasma using the Luminex® 100™ System and the Biotrak ELISA system, respectively. IL-6 and MMP-9 values that were three standard deviations higher than the mean (mean calculated after initial exclusion of any extreme outliers) were excluded prior to analyses.

#### Self-rated health

Self-rated health (SRH) was measured using the first item from the well-known generic health-related quality of life scale, SF-36 (Sullivan et al., 1995; Ware and Sherbourne, 1992): ‘*In general, would you say your health is. . . excellent/very good/good/fair/poor*’. In the following analyses, the item responses were dichotomised into high (excellent/very good/good) and low (fair/poor).

#### Eight-year incident CHD

Incident CHD was defined as fatal or nonfatal myocardial infarction and/or an event of symptomatic angina pectoris requiring invasive coronary revascularisation (percutaneous cardiac intervention or coronary artery bypass graft surgery). CHD data for the LSH study population during the first 8 years from baseline were obtained from the National Causes of Death Register and the National Patient Register, both from the Swedish National Board of Health and Welfare.

### Validation procedure

Construct validity (De Vet et al., 2011) was tested through two hypotheses relating to the reduced scales: (1) the reduced scales would show the same multiple linear regression coefficients regarding biomarkers as the full scales and (2) the reduced scales would receive similar odds ratios to the full scales in logistic regression analyses with SRH (low/high; baseline data) or incident CHD (yes/no; 8-year follow-up data) as the outcome.

All statistical analyses were performed in Stata version 12 (StataCorp, College Station, TX).

The study design was approved by the regional Ethical Review Board, Linköping, Sweden (02-0324). All procedures performed in studies involving human participants were in accordance with the ethical standards of the institutional and/or national research committee and with the 1964 Helsinki declaration and its later amendments or comparable ethical standards.

## Results

Study population characteristics can be seen in Table 1. Six people declined having their data retrieved from the Swedish National Health Data Registries (in the present study this pertained to data on incident CHD), and were excluded from analyses in the present study. The mean age of the study population was 57 years.

**Table 1.** Characteristics of the study population (at baseline unless otherwise stated).

	*n*	%	Mean (SD)
Age	1001	–	57 (11)
Sex	1001	–	
Female	499	50	
Self-rated health	973	–	
Low	261	27	
Biomarkers			
IL-6 (0.54 to 19.52 pg/mL)	903	–	2.0 (2.8)
MMP-9 (2.3 to 166 ng/mL)	903	–	39.1 (25.7)
Incident CHD at 8-year follow-up	59	6	
Psychosocial scales (scores)			
Full scales			
Mastery	943	–	22.5 (3.4)
Self-esteem	942	–	32.2 (4.8)
Sense of coherence, SOC	957	–	68.7 (10.4)
CES-D	932	–	9.0 (7.8)
Vital exhaustion	960	–	30.1 (7.6)
Cynicism	964	–	31.2 (7.7)
Availability of attachment (AVAT)	959	–	5.5 (1.1)
Availability of social integration (AVSI)	957	–	20.5 (5.9)
Reduced scales			
Mastery	943	–	13.3 (2.9)
Self-esteem	942	–	11.4 (2.7)
Sense of coherence, SOC	957	–	30.5 (6.0)
CES-D	932	–	1.6 (2.6)
Vital exhaustion	960	–	2.8 (2.6)
Cynicism	964	–	9.7 (4.4)
Availability of attachment (AVAT)	959	–	3.7 (0.8)
Availability of social integration (AVSI)	957	–	5.5 (2.2)

The full psychosocial scales comprised between six and 20 items, and the reduced scales between three and seven items (Table 2). The longer the full scale, the larger the number of items removed. Cronbach’s alpha values for the full scales varied between 0.75 and 0.93, and for the reduced scales between 0.72 and 0.87. Pearson intercorrelation coefficients between mean values of full and reduced scales varied between 0.86 and 0.98 (Table 2).

**Table 2.** Psychometric properties of and correlations between full and reduced scales (*n* = 932–964).

Domain	Name of scale	Number of items		Range in scale		Range in study		Cronbach’s alpha		Correlation between full and reduced scale mean values
		Full scale	Reduced	Full scale	Reduced	Full scale	Reduced	Full scale	Reduced	
Psychological resources	Mastery^a^	7	6	7–28	0–18	7–28	0–18	0.75	0.72	*r* = 0.98
	Self-esteem	10	5	10–40	0–15	15–40	1–15	0.85	0.84	*r* = 0.92
	Sense of coherence	13	7	13–91	0–42	32–91	7–42	0.82	0.76	*r* = 0.93
Psychological risk factors	CES-D	20	5	0–60	0–15	0–51	0–15	0.86	0.87	*r* = 0.86
	Vital exhaustion	19	5	19–57	0–10	19–56	0–10	0.93	0.87	*r* = 0.93
	Cynicism	12	6	12–60	0–24	12–53	0–22	0.85	0.82	*r* = 0.93
Social support	Availability of attachment (AVAT)	6	4	0–6	0–4	0–6	0–4	0.76	0.76	*r* = 0.91
	Availability of social integration (AVSI)	6	3	6–36	0–9^b^	6–36	0–9^b^	0.85	0.79	*r* = 0.92

a The item removed from Mastery was chosen for reasons of connotation (the Swedish translation was considered too divergent from the meaning of the original item, but the present study did not have the means to validate new translations).

b The reduced scale of AVSI included only four response categories as opposed to six in the full scale.

Construct validity was tested through two hypotheses and, as hypothesised, no significant differences could be found between full and reduced scales in (1) their relationships to biomarkers of stress (Table 3), nor in (2) their relationships to SRH and incident CHD (Table 4). Regression coefficients (Table 3) were in the expected direction (negative regarding psychological resources/social support and positive regarding risk factors), that is, a higher biomarker level is non-beneficial for health. However, for the biomarker IL-6, regression coefficients were all quite low. For AVAT, the coefficient of –0.46 for full scale AVAT is seemingly quite different from +0.07 for the reduced scale regarding MMP-9, but the difference was not statistically significant. Odds ratios (Table 4) were consistently >1 for psychological resources/social support versus higher SRH, as well as for risk factors versus incident CHD; and <1 for risk factors versus higher SRH, as well as for resources/social support versus incident CHD. Cynicism had odds ratios of about 1 versus incident CHD, but this was similar for the full and reduced scales.

**Table 3.** Construct validity tests: Full and reduced scales in relation to IL-6 and MMP-9 (at baseline). Linear regressions adjusted for age and sex. Regression coefficients per SD. Significance tests for full and reduced scales.

Domain	Name of scale	IL-6		*p*-value difference	MMP-9		*p*-value difference
		Full scale	Reduced		Full scale	Reduced	
Psychological resources	Mastery	–0.24 (–0.41; –0.07)	–0.26 (–0.44; –0.08)	0.960	–1.23 (–2.77; 0.31)	–1.29 (–2.88; 0.26)	0.920
	Self-esteem	–0.23 (–0.41; –0.05)	–0.20 (–0.39; –0.02)	0.689	–1.68 (–3.26; –0.12)	–1.67 (–3.23; –0.08)	0.992
	Sense of coherence	–0.20 (–0.38; –0.02)	–0.25 (–0.43; –0.07)	0.726	–2.16 (–3.70; –0.60)	–2.31 (–3.88; –0.77)	0.912
Psychological risk factors	CES-D	0.23 (0.05; 0.41)	0.28 (0.09; 0.46)	0.984	1.82 (0.027; 3.37)	1.56 (–0.05; 3.15)	0.631
	Vital exhaustion	0.36 (0.18; 0.54)	0.37 (0.19; 0.55)	0.968	1.28 (–0.23; 2.85)	0.98 (–0.59; 2.56)	0.849
	Cynicism	0.04 (–0.13; 0.22)	0.03 (–0.15; 0.21)	0.764	2.34 (0.80; 3.88)	2.34 (0.80; 3.91)	0.999
Social support	Availability of attachment (AVAT)	0.03 (–0.14; 0.20)	0.00 (–0.17; 0.17)	0.920	–0.46 (–1.98; 1.05)	0.07 (–1.45; 1.59)	0.548
	Availability of social integration (AVSI)	–0.12 (–0.29; 0.01)	–0.06 (–0.25; 0.10)	0.771	–2.24 (–3.75; –0.75)	–1.82 (–3.35; –0.29)	0.992

IL-6: interleukin-6; MMP-9: matrix metalloproteinase-9.

**Table 4.** Construct validity tests: Full and reduced scales in relation to poor self-rated health (at baseline) and incident myocardial infarction (8-year follow-up). Logistic regressions adjusted for age and sex. Odds ratios per SD. Significance tests for full and reduced scales.

Domain	Name of scale	Poor self-rated health odds ratios		*p*-value difference	Incident myocardial infarction odds ratios		*p*-value difference
		Full scale	Reduced		Full scale	Reduced	
Psychological resources	Mastery	2.03 (1.71; 2.39)	2.02 (1.74; 2.42)	0.872	0.74 (0.56; 0.96)	0.74 (0.56; 0.93)	0.976
	Self-esteem	1.80 (1.54; 2.10)	1.72 (1.50; 2.03)	0.749	0.68 (0.52; 0.89	0.71 (0.55; 0.93)	0.603
	Sense of coherence	1.87 (1.50; 2.19)	1.78 (1.59; 2.17)	0.976	0.81 (0.61; 1.06)	0.78 (0.59; 1.02)	0.872
Psychological risk factors	CES-D	0.47 (0.38; 0.55)	0.44 (0.37; 0.52)	0.984	1.44 (1.12; 1.85)	1.44 (1.14; 1.83)	0.872
	Vital exhaustion	0.34 (0.29; 0.41)	0.35 (0.30; 0.43)	0.741	1.45 (1.10; 1.88)	1.38 (1.05; 1.80)	0.771
	Cynicism	0.85 (0.73; 0.93)	0.83 (0.72; 0.97)	0.992	0.96 (0.72; 1.28)	1.01 (0.76; 1.34)	0.944
Social support	Availability of attachment (AVAT)	1.17 (1.02; 1.34)	1.15 (1.00; 1.32)	0.857	0.83 (0.66; 1.04)	0.84 (0.67; 1.06)	0.802
	Availability of social integration (AVSI)	1.49 (1.30; 1.79)	1.44 (1.23; 1.70)	0.548	0.70 (0.52; 0.94)	0.71 (0.52; 0.96)	0.984

## Discussion

The present study of a middle-aged general population found that it was possible to reduce the number of items in the eight tested psychosocial scales in the given context, using an easily manageable technique, while maintaining the validity of the scales. All relationships found in the construct validity tests were in the directions hypothesised.

The rationale behind the present study was the need, in comprehensive surveys, to be able to investigate several different psychosocial domains even with limited survey space. Previously published results from the LSH study have shown that the psychosocial scales are only lowly to moderately intercorrelated, with domains representing distinctive and complementary constructs. This is also demonstrated by the different relationships found to physical, social and mental dimensions of the SF-36, and to health behaviours. (Festin et al., 2017; Nilsson and Kristenson, 2010; Thomas et al., 2020). However, it would be reasonable to assume that the scales sometimes might be used interchangeable, depending on the scope of the research. To conclude, being able to include several specific psychosocial domains is very important. Choosing only one or two scales could lead to a loss of vital information on other specific psychosocial domains, and using scales as proxy measures for other psychosocial domains could, in the worst case, lead to wrong conclusions.

Several authors have discussed that a large number of items on a scale will not necessarily ensure better psychometric properties, except for high Cronbach’s alpha values, and furthermore that a (very) high Cronbach’s alpha may in fact indicate a high level of item redundancy (Boyle, 1991; Streiner et al., 2015). This may be especially apparent in older scales that were developed when questionnaire length and survey fatigue were not such crucial issues as they are today.

Cronbach’s alphas for all the full scales were above, or even well above, the recommended level of 0.70 for use at group level (De Vet et al., 2011), hence item reduction was an alternative for all scales. Since the mathematical structure of the Cronbach’s alpha algorithm leads to longer scales receiving higher alpha values (De Vet et al., 2011; Sijtsma, 2009), it was expected that values in scales with many items removed would drop the most. This was also the case except for CES-D, where the alpha value even increased slightly. This could be due to the fact that among the discarded items were all of the positively worded items, which we in an earlier study had found to have low correlation to both total CES-D score and to the negatively worded items, as well as to health outcomes (Lundgren et al., 2015). Hence, the removal of these items probably lead to greater homogeneity amongst the remaining negatively worded items. This assumption is supported by the result of analysis of correlation between full and reduces scales after deleting these four items from the full CES-D scale, where the correlation is *r* = 0.90 instead of 0.86.

The item reduction in the present study was performed on a mathematical basis. If the purpose of the present study had been to create official short forms, respondents’ views on what is important for them would have informed the choice of items, giving highest priority to items that respondents find relevant and easy to understand and respond to (Nilsson et al., 2007). However, the purpose of the present study was to use a method that is easily available to most researchers.

A limitation of the present study is that the respondents only answered full scales. There is a risk that respondents will interpret and answer individual items in a scale differently in the presence or absence of other items. The study design did not give the respondents the chance to answer any reduced scales per se, that is, the present study does not allow for evaluations of possible context effects. Nor does it allow for a test-retest (reliability test) or a test of longitudinal validity (responsiveness, ability to capture all relevant changes). As the context of the present study is psychosocial determinants for CHD by the means of a multipurpose survey and use of data at group level, the validity of using reduced scales for other contexts has not been tested, especially not for use of data at an individual level. While the present study group, drawn from the general population, is a relatively healthy population (as general populations often are), the distribution of scale scores in the study population still covers the possible scale score distributions fairly well (Table 2). One final limitation might be that the generally low regression coefficients for both full and reduced scales regarding IL-6 make it difficult to draw firm conclusions.

A strength of the present study is the range and high number of characteristics and variables in the LSH cohort which makes this very beneficial for validation studies, especially the possibility of using not only other self-report data (SRH), but also biomedical data which are cross-sectional (IL-6, MMP-9) as well as prospective (CHD outcome 8 years later).

## Conclusion

In conclusion, we found that it was possible to reduce the number of items in psychosocial scales without threatening the validity for use at group level in population surveys, using an easily manageable technique.

See supplementary material for Measuring psychosocial factors in health surveys using fewer items

## Supplemental Material

sj-pdf-1-hpo-10.1177_2055102920975983 – Supplemental material for Measuring psychosocial factors in health surveys using fewer items Click here for additional data file. Supplemental material, sj-pdf-1-hpo-10.1177_2055102920975983 for Measuring psychosocial factors in health surveys using fewer items by Evalill Nilsson, Peter Garvin, Karin Festin, Marika Wenemark and Margareta Kristenson in Health Psychology Open
