# Supplementary material for: Measuring psychosocial factors in health surveys using fewer items
Source: Health Psychol Open. 2020 Dec 24;7(2):2055102920975983. doi: 10.1177/2055102920975983 (PMC7768575; doi:10.1177/2055102920975983)
Supplement: sj-pdf-1-hpo-10.1177_2055102920975983 – Supplemental material for Measuring psychosocial factors in health surveys using fewer items [file sj-pdf-1-hpo-10.1177_2055102920975983.pdf]

Measuring psychosocial factors in health surveys using fewer items

Evalill Nilsson, Peter Garvin, Karin Festin, Marika Wenemark, Margareta Kristenson

Supplemental table 1. Items in psychological resources scales.

Remaining and discarded (in italics) items from Pearlin's Mastery Scale after reduction

**Item**	**Response categories and scoring**			
To what extent are the following statements accurate for you?	*Not at all*	*To a minor extent*	*To some extent*	*To a major extent*
There is really no way I can solve some of the problems I have.	3	2	1	0
I have little control over the things that happen to me.	3	2	1	0
I can do just about anything I really set my mind to.	0	1	2	3
I often feel helpless in dealing with the problems of life.	3	2	1	0
What happens to me in the future mostly depends on me.	0	1	2	3
There is little I can do to change many of the important things in my life.	3	2	1	0
Sometimes I feel that I'm being pushed around in life.				

Remaining and discarded (in italics) items from Rosenberg's Self-Esteem Scale after reduction

**Item**	**Response categories and scoring**			
To what extent are the following statements accurate for you?	*Not at all*	*To a minor extent*	*To some extent*	*To a major extent*
I take a positive attitude toward myself.	0	1	2	3
All in all, I am inclined to feel that I'm a failure.	3	2	1	0
I certainly feel useless at times.	3	2	1	0
At times I think I am no good at all.	3	2	1	0
On the whole, I am satisfied with myself.	0	1	2	3
I feel that I'm a person of worth, at least on an equal plane with others.				
I feel that I have a number of good qualities.				
I am able to do things as well as most other people.				
I feel I do not have much to be proud of.				
I wish I could have more respect for myself.				

Remaining and discarded (in italics) items from Sense of Coherence-13 after reduction

Considering the last couple of months:	*Left endpoint*						*Right endpoint*
Doing the things you do every day is… [A source of deep pleasure and satisfaction] vs. [A source of pain and boredom]	6	5	4	3	2	1	0
Has it happened that people whom you counted on disappointed you? [It has never happened] vs. [Many times]	6	5	4	3	2	1	0
Do you have the feeling that you are in an unfamiliar situation and don't know what to do? [All the time] vs. [Very seldom or Never]	0	1	2	3	4	5	6
Has it happened in the past that you were surprised by the behaviour of people whom you thought you knew well? [It has never happened] vs. [Many times]	6	5	4	3	2	1	0
Does it happen that you have feelings inside you would rather not feel? [All the time] vs. [Very seldom or Never]	0	1	2	3	4	5	6
How often do you have the feeling that there's little meaning in the things you do in your daily life? [All the time] vs. [Very seldom or Never]	0	1	2	3	4	5	6
Until now your life has had… [No clear goals or purpose at all] vs. [Very clear goals and purposes]	0	1	2	3	4	5	6
Do you have the feeling that you don't really care about what goes around on you? [Very seldom or Never] vs. [All the time]							
Do you have the feeling that you are treated unfairly? [All the time] vs. [Very seldom or Never]							
Do you have very mixed-up feelings and ideas? [All the time] vs. [Very seldom or Never]							
Many people – even those with a strong character – sometimes feel like sad sacks (losers) in certain situations. How often have you felt this way in the past? [Never] vs. [All the time]							

When something happened, have you generally found that…? [You overestimated or underestimated its importance] vs. [You saw things in the right proportions]

How often do you have feelings that you are not sure you can keep under control? [All the time] vs. [Very seldom or Never]

Supplemental table 2. Items in psychological risk factor scales.

Remaining and discarded (in italics) items from CES-D after reduction

Item	Response categories and scoring			
During the past week:	*<1 day*	*1-2 days*	*3-4 days*	*5-7 days*
I felt that I could not shake off the blues even with help from my family or friends.	0	1	2	3
I felt depressed.	0	1	2	3
I felt that everything I did was an effort.	0	1	2	3
I felt sad.	0	1	2	3
I could not get "going".	0	1	2	3
I was bothered by things that do not usually bother me.				
I did not feel like eating; my appetite was poor.				
I had trouble keeping my mind on what I was doing.				
I thought my life had been a failure.				
I felt fearful.				
My sleep was restless.				
I talked less than usual.				
I felt lonely.				
People were unfriendly.				
I had crying spells.				

I felt that people dislike me.
I felt I was just as good as other people.
I felt hopeful about the future.
I was happy.
I enjoyed life.

Remaining and discarded (in italics) items from Vital Exhaustion after reduction

**Item**	**Response categories and scoring**		
Considering the last couple of months	*Never*	*Sometimes*	*Often*
Do you feel more listless recently than before?	0	1	2
Do you have the feeling that you can't cope with everyday problems as well as you used to?	0	1	2
Do you sometimes feel like your body is like a battery losing its power?	0	1	2
Do you feel downcast?	0	1	2

Have you ever had a feeling lately, like “I do not achieve enough. I could achieve more if only I were healthier, not so weak, not so limp”?	0	1	2
Do you often feel tired?			
Have you felt less confident lately?			
Do you have a feeling that you haven’t accomplished much lately?			
Do you believe that you have come to a “dead end”?			
Do you feel like you are losing your self-restraint?			
Have you noticed lately that it takes a longer time than before to “get going”?			
Do you lately think more often about acquaintances or relatives that are deceased?			
Do you have a feeling that nobody can help you with those problems deep inside?			
Are you becoming less satisfied with yourself?			
Do you feel less capable of doing something useful these days?			
Do minor hassles irritate you easily these days?			
Would you want to be dead at times?			
Can you bring yourself less and less to leave the house and go for a visit?			
Do you have the feeling these days that you don’t have what it takes anymore?			

Remaining and discarded (in italics) items from Cook-Medley Cynicism Scale after reduction

**Item**					
To what extent do you agree with the following statements?	*Not at all*	*To a minor extent*	*Neither nor*	*To some extent*	*To a major extent*
I think most people would lie to get ahead.	0	1	2	3	4
Most people will use somewhat unfair means to get profit or an advantage rather than to lose it.	0	1	2	3	4
Most people make friends because friends are likely to be useful to them.	0	1	2	3	4
Most people inwardly dislike putting themselves out to help other people.	0	1	2	3	4
I have often met people who were supposed to be experts who were no better than I.	0	1	2	3	4
People generally demand more respect for their own rights than they are willing to allow for others.	0	1	2	3	4
I have often had to take orders from someone who did not know as much as I did.					
I think a great many people exaggerate their misfortunes in order to gain the sympathy and help of others.					
It takes a lot of argument to convince most people of the truth.					
Most people are honest chiefly through fear of being caught.					
No one cares much what happens to you.					
It is safer to trust nobody.					

Supplemental table 3. Items in social support scales.

Remaining and discarded (in italics) items from Availability of Social Integration after reduction

Item	Response categories and scoring			
	None	1-2	3-5	6 or more
Number of friends who at any time can come and visit your home and you would not be embarrassed if it were untidy.	0	1	2	3
Number of friends or family members with whom you can talk frankly.	0	1	2	3
Someone available – apart from family – to whom you can turn in times of difficulties.	0	1	2	3
Number of people with whom you share interests.				
Number of people met during an ordinary week.				
Someone available whom you can ask small favors.				

The reduced scale included only four response categories as opposed to six in the full scale.

Remaining and discarded (in italics) items from Availability of Attachment after reduction

Item	Response categories and scoring	
	No	Yes
Someone special whom you can lean on?	0	1
Someone who feels very close to you?	0	1

Someone to share your feelings with?	0	1
Someone to confide in?	0	1
Someone to hold and comfort you?		
Someone at home who really appreciates what you do for him/her?		
